# Supporting Effective Peer Review at *Investigative Ophthalmology & Visual Science*

**DOI:** 10.1167/iovs.66.4.79

**Published:** 2025-04-29

**Authors:** Abigail T. Fahim, Joseph Carroll

**Affiliations:** 1 Department of Ophthalmology and Visual Sciences, University of Michigan, Ann Arbor, Michigan, United States; 2 Council of Vision Editors Fellowship Program, Washington, DC, United States; 3 Department of Ophthalmology & Visual Sciences, Medical College of Wisconsin, Milwaukee, Wisconsin, United States; 4 Editor-in-Chief, *Investigative Ophthalmology & Visual Science*

Rigorous peer review remains an important and indispensable step in the scientific publication process. However, peer reviews have become more challenging to obtain as the demand has outpaced the supply. As shared in a previous editorial,^1^ there has been significant growth in the number of peer-review invitations sent by *Investigative Ophthalmology & Visual Science (IOVS)* over the past few years. In 2024 alone, we sent out over 8000 reviewer invitations! There are several underlying factors that contribute to this surprising growth and unprecedented number of invitations. First, there has been a substantial increase in the number of submitted articles, which speaks to the standing of *IOVS* among the vision research community (*IOVS* had the second highest h-index among ophthalmology and vision science journals in 2024). Second, many invitations are either declined or go unanswered, thereby increasing the number of invitations per article (for 2024 this was 7.5 invites per article). Although this trend is not unique to *IOVS*,^2^ we must be proactive and take steps to mitigate this growing challenge. Inaccuracies in the *IOVS* reviewer database might account for some instances of “no response” and we are making strides to cull and update the reviewer database. Furthermore, our reviewers are stretched very thin and receive numerous review invitations. Understandably, when faced with other obligations and deadlines, peer review falls further and further down the list of priorities. For some junior scientists, an additional contributing factor may be intimidation by the peer review process due to a lack of experience. This last factor can be addressed by education to provide guidance and instill confidence for those with little peer review experience. Given the complexity of the issue, and given that guidelines vary by journal,^3^ we wanted to provide an editorial to help support effective peer review of articles submitted to *IOVS*, with a focus on those readers who may not have extensive peer review experience.

## Effective Review Approach

There are many instructive resources available for both writing and reviewing manuscripts effectively.^4^^–^^9^ In many ways, the guidance on how to *write* a manuscript can be used to guide the *review* of a manuscript. Anyone new to the peer-review system should consult these resources, just as they might consult prior studies when trying to learn a new method in the laboratory. To complement these resources, we want to highlight some specific areas valued by *IOVS* and to clarify some frequently asked questions received from current and prospective reviewers.^1^

**Figure 1.**
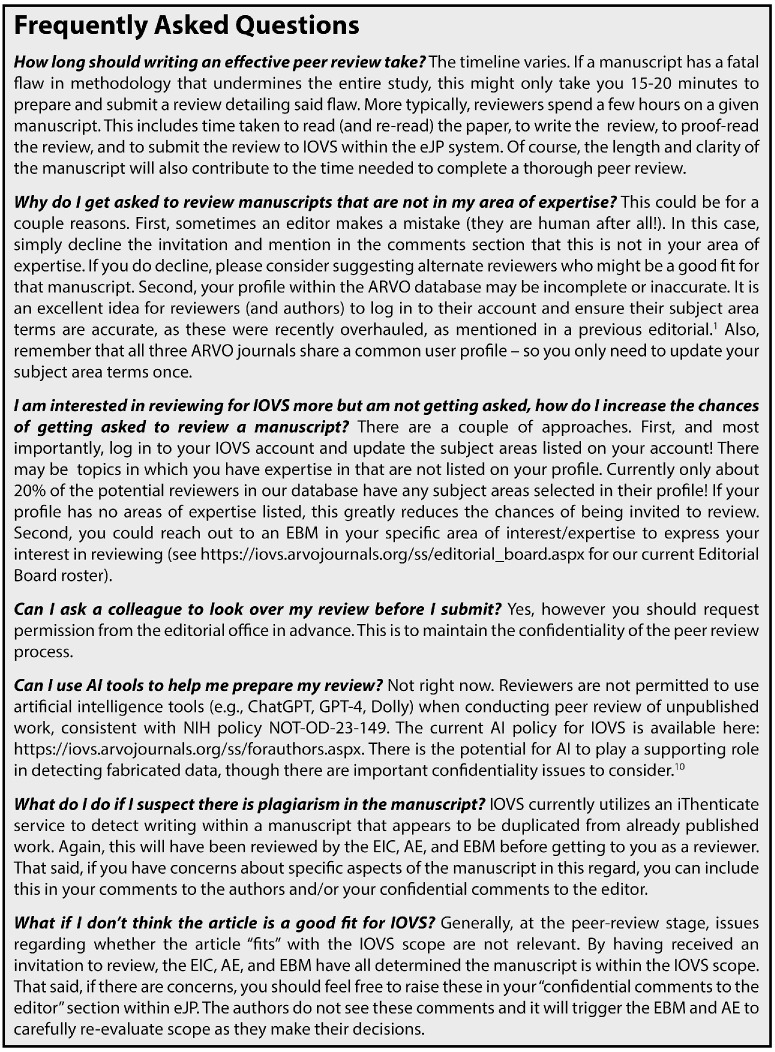
Frequently asked questions about peer review at *Investigative Ophthalmology & Visual Science*.

It is important to start by acknowledging that there is not one “right” way to review a manuscript. If you have a system that works well for you, use it. Even better, share it with others so they can learn! For reviewers looking for guidance, a generally effective approach is to read the manuscript at least twice. From the first read, the reviewer quickly learns the overall gist of the manuscript. The reviewer might make minor notes, highlight key sentences and sections, or identify parts that seem unclear. The goal is to leave this first reading with an idea of the overall purpose of the study and general impression of the manuscript. If a major methodological flaw is identified in this first reading, a second reading may be unnecessary, and the reviewer can proceed directly to preparing comments based on the major flaw in the study.

The second read is much more thorough and provides an opportunity to take more detailed notes and to begin drafting comments that will be included in the review. It is important to look closely at all elements of the manuscript, especially the tables and figures. It may be helpful to write comments in a word processing document, from which they can be copied and pasted into the *IOVS* online reviewer fields before submission. This is just one approach, and reviewers should find an approach that works best for them.

## Review Structure

Effective reviews typically start with a *brief* synopsis of the manuscript, including a high-level overview of the purpose, approach, and/or key findings of the study. The synopsis should be a short paragraph (3–5 sentences) and not a detailed summary. This useful element helps orient the reviewer prior to making more detailed comments and may also assist the editors (and even authors) in cases where there may be a disconnect between what the reviewer understood and what the authors intended.

Many think the purpose of peer review is simply to point out what is “wrong” with the manuscript, and this is often the focus of the reviews we receive at *IOVS*. However, the review is not just a guide to the authors for revision but will also be used by the editorial board member (EBM) and associate editor (AE) to help them decide whether the manuscript should be published. To support this decision-making process, an effective review should also include perceived strengths of the manuscript. Generally, these comments are best presented as a separate section (usually at the beginning of the review), however, there are cases where one might choose to discuss the strengths and weaknesses of specific aspects of the manuscript together.

As for the comments, critiques, and suggestions to the authors, we sometimes see these grouped or categorized as “major” and “minor” issues. This is an excellent approach and helps the EBM and AE understand the gravity of each point and thus helps the authors prioritize and focus their revision (if the eventual decision includes an opportunity for revision). A key aspect of these areas for improvement is to be as specific as possible. In addition, do not only point out the weaknesses, but also indicate what it would take to remedy the identified problems. The time and effort required to address an issue can often be the difference between a reject decision or a major revision decision. Furthermore, specifying how weaknesses could be addressed will help the authors understand what is expected to achieve a publishable manuscript and avoid wasted time and effort, in the event they are offered an opportunity to revise.

The entire manuscript should be reviewed, including the abstract, introduction, methods, results (including tables and figures), and discussion. It is important to not neglect the supplementary files, which often contain important methods or data. Additionally, an effective review will comment on the quality of the presentation and provide an overall impression of the importance/significance of the study. Here are a few key things to consider when assessing these aspects of the manuscript.

***Abstract*** – Does the abstract accurately reflect what is presented in the manuscript?

***Introduction*** – Do the authors effectively and succinctly convey the knowledge gap being addressed (i.e. the need and motivation for the present study)?

***Methods*** – Are the methods and experimental approach appropriate to address the main research question? Are the methods presented with sufficient detail to enable replication by others? Are statistical methods appropriate and is the study appropriately designed?

***Results*** – Are the results comprehensive and presented in a logical order? Are the data presented transparently or are key data missing from the manuscript? Are the figures clear and easy to interpret? Are there figures or tables that could be relegated to Supplementary Content to improve the overall readability of the manuscript? Are there items in the Supplementary Content that should be presented in the main manuscript? Note that issues related to figures and data presentation can be part of the overall “presentation” assessment (see below). Finally, are the results presented with adequate scientific rigor? Consider how many times the experiments were performed, the sample sizes, whether appropriate controls were included, and whether the data are sufficiently quantitative vs qualitative for the questions being asked. These considerations are related to methods and statistics (above) but go beyond those questions to evaluate the quality of the data.

***Discussion*** – Are the conclusions supported by the data? Do the conclusions have significant impact and is the significance reasonably and accurately discussed? Are the results put into context with prior results on the topic or linked with related findings in an integrative fashion? Are key limitations of the study disclosed and discussed?

***Presentation*** – Is the writing clear? This does not mean the reviewer should endeavor to copyedit the manuscript. This is not what we are looking for in an effective peer review, however, we do get many reviews where the reviewer simply provides a list of grammatical corrections. All accepted *IOVS* articles will undergo professional copyediting prior to publication, so we are not looking for reviewers to focus on minor issues of grammar. Of course, a reviewer can and should point out issues with writing if they interfere with the clarity of the presentation or the interpretation of results. But an exhaustive list of copyedits is not needed. If there are significant issues, a single review comment such as “*The authors should carefully edit the next revision for grammar and clarity, engaging with formal copyediting support as needed.*” or “*The introduction lacks clarity and does not clearly motivate the current study.*” would be sufficient.

***Overall*** – Does the manuscript address an important problem in the field and/or represent a significant advance in knowledge? This is sometimes difficult to quantify, but editors value your overall impression of the significance of the study. This assessment may require perusal of prior literature, especially major references from the manuscript, to determine whether the submitted manuscript provides new valuable information that differs significantly from prior studies.

When submitting the review, the reviewer is asked to recommend whether the manuscript should receive a decision of “Reject,” Major Revisions,” “Minor Revisions,” or “Accept.” Note that this recommendation should not be included in the comments to the authors, only through using the drop-down menu in the online submission site. On balance, the written comments (the perceived strengths and weaknesses of the manuscript) should align with the recommendation. In other words, do not provide a long list of fatal flaws and then recommend “Accept,” or provide nothing but praise and one small item for improvement and then recommend “Reject.” It is important that the recommendation is supported by the comments.

A seldom used feature of the *IOVS* peer-review system is the “confidential comments to editors” section. This is a place where the reviewer can provide “color” or additional context for the review. For example, perhaps the reviewer does not have expertise in a specific area and thus did not review that aspect of the paper as thoroughly as other parts. This could be shared in the confidential comments: “*Note that I do not have expertise in statistics, so the editors may wish to have someone with statistical expertise review and comment on the analysis performed in Figure 3.*” Alternatively, if the reviewer was wavering between a “reject” and “major revisions” recommendation, these confidential comments to the editors could provide context as to justify the chosen recommendation. Additionally, these comments could be used to disclose to the editors potential conflicts of interest you might have.

## Review Etiquette: Be Professional and Be Kind

Effective reviews are specific, on time, and professional. I (author and editor J.C.) once received a review on a manuscript I submitted to *IOVS* that included the following comment: “*This paper was written in an extremely sloppy manner. It was a burden to read and review. I usually look forward to learning something new from a paper.*” Certainly not overly constructive. Perhaps now more than ever, it is important that the scientific community leads by example with respect to civil communication. This is why we suggest entering initial reviewer comments in a word processing document as opposed to typing directly into the submission system, as it facilitates proofreading and editing comments – both for clarity and civility. Editors at *IOVS* have had to redact some very hurtful and irrelevant comments from reviewers, and we will continue to do so (although we cannot catch everything). Editors have also reached out directly to reviewers who have submitted rude or demeaning comments. An effective review is one in which all comments are relevant to the manuscript, constructive, and not personal attacks on the author(s). Whereas no one is perfect (nor expected to be), it is important that as peers we endeavor to be better when it comes to reviewing one another's science. We hope *IOVS* is a leader in this effort in the coming years.

## Summary

In closing, the take home points when being asked to review at *IOVS* are to (1) respond to the invitation as soon as possible (accept or decline), (2) if you accept, be on time and proofread your review before submitting, (3) utilize this guide and other published resources to sharpen review skills and confidently accept review invitations, and (4) be respectful in your review (and in your communications with *IOVS* editors and staff). There have been some recent improvements to our review functionality and there will be growth in our Reviewer-in-Training program to help cultivate the next generation of peer reviewers at *IOVS* (more on this later in 2025). In the meantime, we encourage our readers to think of junior colleagues and trainees who might be good peer reviewers and support their growth and training in peer review. Together we can increase the pool of skilled and willing peer reviewers to meet the growing demand.
